# Plant Growth Promoting of Endophytic *Sporosarcina aquimarina* SjAM16103 Isolated from the Pneumatophores of *Avicennia marina L.*

**DOI:** 10.1155/2012/532060

**Published:** 2012-07-03

**Authors:** S. Rylo Sona Janarthine, P. Eganathan

**Affiliations:** ^1^Faculty of Marine Science, Annamalai University, Chidambaram 608 502, India; ^2^Biotechnology Division, M S Swaminathan Research Foundation, Chennai 600 113, India

## Abstract

Endophytic *Sporosarcina aquimarina* SjAM16103 was isolated from the inner tissues of pneumatophores of mangrove plant *Avicennia marina* along with *Bacillus* sp. and *Enterobacter* sp. Endophytic *S. aquimarina* SjAM16103 was Gram variable, and motile bacterium measured 0.6–0.9 **μ**m wide by 1.7–2.0 **μ**m long and light orange-brown coloured in 3-day cultures on tryptone broth at 26°C. Nucleotide sequence of this strain has been deposited in the GenBank under accession number GU930359. This endophytic bacterium produced 2.37 **μ**Mol/mL of indole acetic acid and siderophore as it metabolites. This strain could solubilize phosphate molecules and fixes atmospheric nitrogen. Endophytic *S. aquimarina* SjAM16103 was inoculated into four different plants under *in vitro* method to analyse its growth-promoting activity and role inside the host plants. The growth of endophytic *S. aquimarina* SjAM16103 inoculated explants were highly significant than the uninoculated control explants. Root hairs and early root development were observed in the endophytic *S. aquimarina* SjAM16103 inoculated explants.

## 1. Introduction

The genus *Sporosarcina*, which belongs to the family Bacillaceae, was created by Kluyver and van Neil [1] to accommodate bacteria that have spherical or oval-shaped cells, low DNA G+C content (40–42 mol%), and MK-7 as the major menaquinone. *Sporosarcina* species can be differentiated from other members of the Bacillaceae, by their coccoid or rod-shaped cells, motility, sporulation, and possession of MK-7 as the major menaquinone and A4*α* as the peptidoglycan variant. The genus *Sporosarcina* originally comprised two species, *S. ureae* and *S. halophila* [2, 3]. However, three species of the genus *Bacillus*, namely, *Bacillus globisporus* [4], *B. psychrophilus* [5], and *B. pasteurii* [6], which belonged to rRNA group 2 [7] and contain L-lysine in their cell wall, have recently been transferred to the genus *Sporosarcina* [8] as *S. globispora*, *S. psychrophila*, and *S. pasteurii* also identified a novel species, *S. aquimarina*  isolated from seawater. However, there were no published reports on endophytic *S. aquimarina*  isolated from the living cells. Therefore, the present study was aimed to investigate the role and effects of endophytic *S. aquimarina* isolated from the pneumatophores of *A. marina*. 

Endophytic bacteria are living inside the plant tissue without eliciting symptoms of disease, common to a large number of plant species. Endophytic bacteria can promote the plant growth and yield and can act as a biocontrol agent [9]. In recent years, much attention has been paid to natural methods of crop growing in expectation of moving toward agriculturally and environmentally sustainable development [10]. Endophytic bacteria promote plant growth due to their abilities in nitrogen fixation [11], phytohormone production [12], solubilization of phosphorus [13], and disease control [14, 15]. 

Endophytic microbial inocula, primarily bacteria, are used as propagule priming agents, both as *in vitro* cocultures and transplanting [16]. It is an emerging trend in biotechnological approach aimed at reducing chemical input in plant production, while increasing plant fitness, productivity, and resistance to diseases in the context of sustainable horticulture.

In the present study, endophytic bacteria were isolated from the surface sterilized pneumatophores of *A. marina*. The isolates were identified phenotypically and genotypically. The isolate *S. aquimarina* was taken for further investigation. This endophytic bacterium was inoculated into four different plants, two fresh water plants (*Bacopa monnieri* and *Eupatorium triplinerve*) and two mangrove plants (*Excoecaria agallocha* and *A. marina*) to analyse its growth-promoting efficacy and role as endophyte. These plants were selected based on their needs in the society, because they have high medicinal and economical values. Medicinally, they are used for curing skin diseases, even for leprosy [17], HIV [18], fungal diseases [19], mental disorders [20, 21], and economically as fire wood, match boxes, paper pulp [17], and used as fodder in India and in Australia. 

## 2. Materials and Methods

### 2.1. Isolation of Strains and Growth Condition

Endophytic bacterium was isolated from the inner tissues of healthy pneumatophores. Parts of pneumatophores about 1 cm of diameter were sterilized with 70% ethanol and 0.1% mercuric chloride [22]. Sterilized parts were excised with a sterile scalpel blade. Slices (0.1 cm thickness) were placed on nutrient agar plates and incubated at 26°C for 48 h. Bacterial growth associated with the pneumatophore sections was purified by repeated plating on nutrient agar, and cultures were maintained as spore suspensions by freezing in 20% (V/V) glycerol.

### 2.2. Phenotypic Characterization

The isolate was Gram-stained and examined microscopically for its morphological characteristics. Some set of physiological characteristics include acid production from sugars (TSI), sodium citrate utilization, urease production, starch, gelatin hydrolysis, and voges-proskauer reaction was carried out using standard protocols described by Gordon et al., [23]. Casein hydrolysis was detected after 3 days of incubation on nutrient agar supplemented with 2% skimmed milk. Growth in the presence of NaCl (2%, 3%, 5%, 7%, and 9%) was determined in nutrient broth as the basal medium during incubation at 28°C for 3 days. It also was estimated at selected temperatures (2, 4, 6, 8, 10, 26, 32, 40, and 50°C) and pH (4.5, 5.5, 7.0, and 9.0) in agar slants incubated at 28°C for 3 days.

### 2.3. ****16S rRNA Gene Sequence Analysis

Genomic DNA was isolated from pure culture [24]. A large fragment (800–1100 bp) of 16S rRNA was amplified by PCR using primers 5′-TGA GGA AGA TAA TGA CGG-3′ and 5′-CCT CTA TCC TCT TTC CAA CC-3′. The 50 *μ*L PCR reaction mixtures contained 100 ng of DNA extract, 1 × Taq reaction buffer, 20 pmol of primers, 200 *μ*M dNTPs, and 1.5 U of Taq DNA polymerase (Promega). The thermocycling conditions consisted of an initial denaturation at 94°C for 3 mins, 30 amplification cycles of 94°C for 1 min (denaturation), 57°C for 1 minute (annealing), 72°C for 2 mins (extension), and final polymerization at 72°C for 4 mins. PCR product was purified and sequenced. Searches in the GenBank/EMBL/DDBJ/PDB data libraries were performed using BLAST (blastn) search algorithm [25] in order to establish the identity of the isolate.

### 2.4. Determination of Cellular Fatty Acid Composition

Cellular fatty acids composition of endophytic bacterium was analyzed using the Sherlock system (MiDi Company, USA) and according to the manufacturer's instructions. 

### 2.5. Plant Growth Promoting Activities 

#### 2.5.1. IAA Production

Indoleacetic acid (IAA) produced by bacterium was assayed colorimetrically using FeCl_3_-HClO_4_ [26]. The bacteria were grown in modified nutrient broth M26 for 24 hours on a gyratory shaker (150 rpm) at room temperature as seed culture. The medium contained (in 1000 mL distilled water) 5 g NaCl, 10 g peptone, and 10 g of beef extract. After overnight incubation, 100 *μ*L of culture was inoculated to 10 mL minimal salt (MS) medium amended with 5 mML tryptophan [27] and grown again for 48 hours on the shaker. The MS medium contained (in 1000 mL distilled water) 1.36 g KH_2_PO_4_, 2.13 g Na_2_HPO_4_, 0.2 g MgSO_4_·7H_2_O, and trace elements. The pH of MS medium was adjusted to 7.0 before autoclaving. L-Tryptophan solution was prepared as stock solution containing (in 100 mL distilled water) 10 g glucose, 1 g glucose, 1 g L-Tryptophan, and 0.1 g yeast extract. The stock solution was filtered through a sterile 0.2 *μ*m membrane filter (Millipore). 1.5 mL bacterial broth culture was centrifuged at 12,000 rpm for 5 mins. One millimeter of the supernatant was added to 2 mL FeCl_3_-HClO_4_ reagent. After 25 mins (after color density reaches its maximum), the mixture was read in UV spectrophotometer at 530 nm absorbance. The amount of IAA produced per milliliter of culture was estimated using a standard curve. The number of bacterial population in the culture expressed in colony forming unit (CFU) was estimated by the Miles and Misra drop plate method.

#### 2.5.2. Phosphate Solubilization

Phosphate solubilization test was conducted qualitatively by plating the bacterium in agar containing precipitated tricalcium phosphate. The medium was a modification of Pikovskaya medium [28]. 

#### 2.5.3. Nitrogen Fixation

Fixation of atmospheric nitrogen by the bacterium was tested qualitatively using Burk's N-free medium [28]. 

#### 2.5.4. Sulphur Reduction

Reduction of sulphur by the bacterium was tested qualitatively by sulphate API agar [28].

#### 2.5.5. Siderophore Production

Siderophore production was tested qualitatively using chrome azurol S (CAS) agar as described by Alexander and Zuberer [29]. The bacterial culture was spread on the CAS agar plates with three replications. Orange halos around the colonies after overnight incubation indicated siderophore production.

### 2.6. Inoculation of Explants with Endophytic Bacterium

Nodal segments (length: 0.5 cm) of four different plants (*Bacopa monnieri, Eupatorium triplinerve, Excoecaria agallocha*, and *Avicennia marina*) were disinfected by sonicating in water for 20 min and dipping in 70% ethanol for 1 min, followed by 25 min of sodium hypochlorite (25%)/Tween 80 (0.01%) solution and rinsed three times with distilled and sterile water [30]. 

The sterile explants of *Bacopa monnieri *and* Eupatorium triplinerve* were cultured in a hormone-free Murashige-Skoog (MS) medium [31] with the addition of 200 *μ*L of endophytic *Sporosarcina aquimarina* SjAM16103. The sterile explants of *Excoecaria agallocha* and *Avicennia marina* were cultured in a hormone-free X medium (M S Swaminathan Research Foundation, India) with the addition of 200 *μ*L of endophytic *Sporosarcina aquimarina* SjAM16103.

Then, these explants were cultured under a photoperiod of 16 h of light and 8 h of dark under an irradiance of 52 mmol m^−2^ seg^−1^. The explants without endophytic *Sporosarcina aquimarina* SjAM16103 were marked as control explants. 

### 2.7. Statistical Analysis

The whole experiment was set up in the randomized design with 10 replicas. All the data collected from these experiments were subjected to an analysis of variance (ANOVA) using SPSS statistical tool. The significant level (*P* > 0.05) was evaluated between various growth parameters (shoot length, number of roots, and root length).

## 3. Results 

### 3.1. Morphology

**Figure 1:**
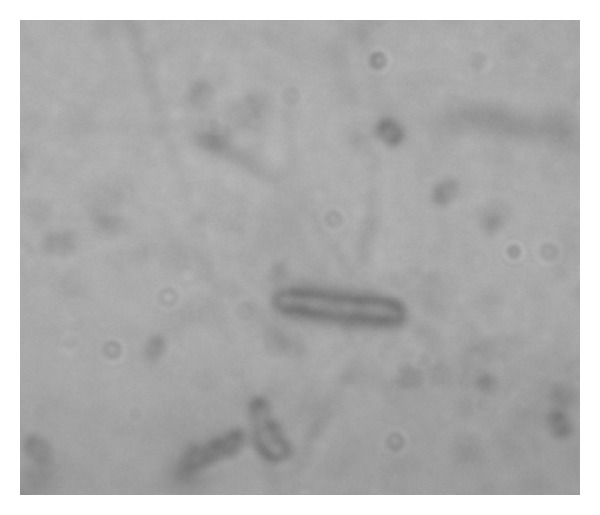
*Sporosarcina aquimarina* SjAM16103.

During this study, 13 bacterial strains were isolated from the pneumatophores of *A*. *marina L*. Among them, four strains (SjAM16101, SjAM16102, SjAM16103, and SjAM16104) were genotypically analysed as *Bacillus* sp., *Enterobacter* sp., *Sporosarcina aquimarina*, and *Bacillus cereus*, respectively. Strain SjAM16103 was Gram variable, and motile bacterium measured 0.6–0.9 *μ*m wide by 1.7–2.0 *μ*m long (Figure 1) and light orange-brown coloured in 3-day cultures on tryptone broth at 26°C. 

### 3.2. Phenotypic Characteristics

**Figure 2:**
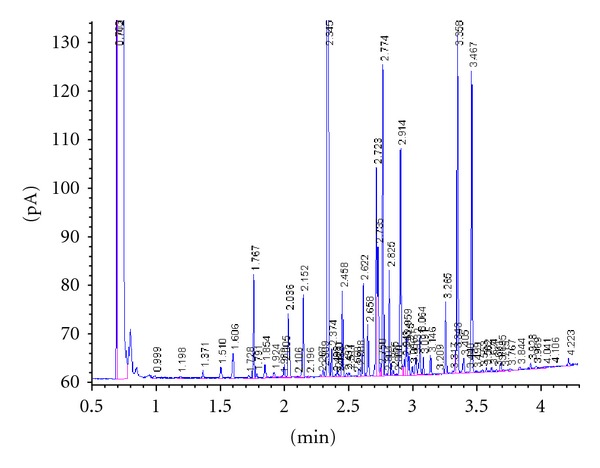
Gas chromatographic methyl ester profiles of endophytic *Sporosarcina aquimarina* SjAM16103.

Phenotypic characteristics of strain SjAM16103 are given in Table 1. The optimal growth temperature was 32°C. Strain SjAM16103 grew at 26–50°C but not at 2–10°C. The optimal pH for growth was 7.0, and growth was inhibited at pH values below 5.0. Strain SjAM16103 grew in the presence of 2–9% NaCl. Gelatin was hydrolysed and showed catalase, urease, and oxidase activities. Acid was produced in the triple sugar iron test. Fatty acids compositions of strain SjAM16103 are given in Table 3. Gas chromatographic methyl ester profiles of strain SjAM16103 are given in Figure 2.

### 3.3. ****16S rRNA Gene Sequences

The 16S rRNA of strain SjAM16103 was directly sequenced following PCR amplification, and its partial nucleotide sequence was determined. The 16S rRNA sequence of strain SjAM16103 was 971 bp long (Table 2) and was identified as *Sporosarcina aquimarina* with highest similarity value of 98%. The nucleotide sequence of *S. aquimarina * SjAM16103 has been deposited in the GenBank/EMBL/DDBJ/PDB under accession number GU930359.

### 3.4. Plant Growth Promoting Activities

Endophytic *S. aquimarina* SjAM16103 produced 2.37 *μ*Mol/mL of IAA. This strain could solubilize phosphate molecules and fix atmospheric nitrogen. Orange halos around *S. aquimarina* SjAM16103 colonies were observed. This confirmed that *S. aquimarina* SjAM16103 was a siderophore producing bacteria (Table 4).

### 3.5. Inoculation of Explants with *S. aquimarina* SjAM16103

Growth parameters were measured to assess the growth promotion capability of endophytic *S. aquimarina* SjAM16103 in four different explants. The numbers of roots were counted. Shoot length and root length were measured in centimeters. The measurements were recorded; for 30–35 days in fresh water plants *B*. *monnieri* and *E*. *triplinerve* and for 35–45 days in mangrove plants *E*. *agallocha* and *A*. *marina*. 

#### 3.5.1. In *B. monnieri L.*

**Figure 3:**
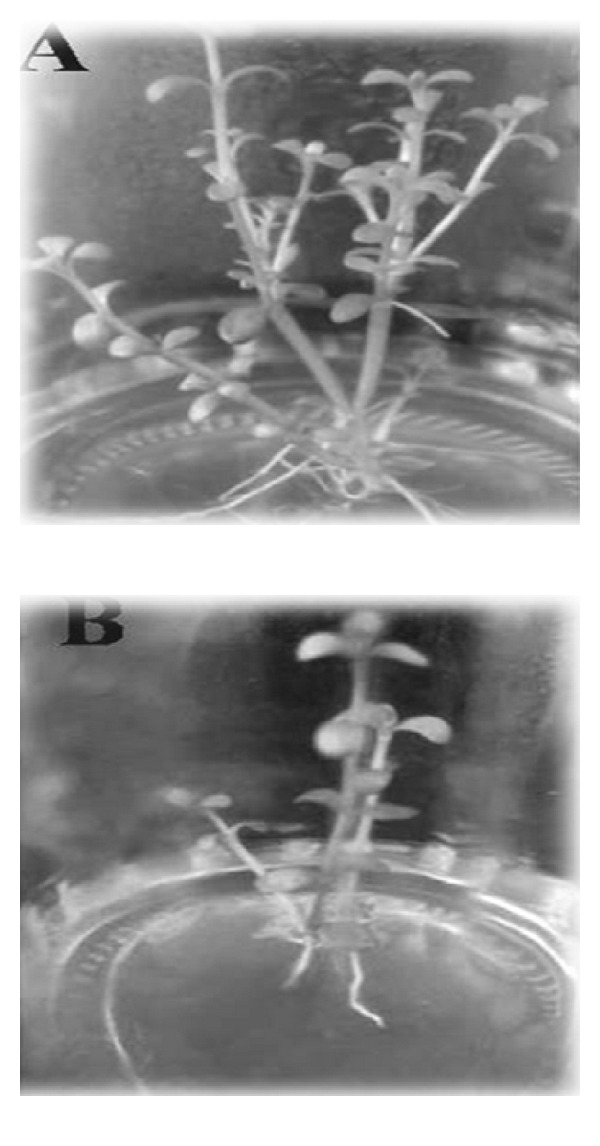
Inoculation of endophytic *S. aquimarina* SjAM16103 in *B. monnieri* (*in vitro*). (A) *S. aquimarina* SjAM16103 inoculated explants; (B) control explants.

**Figure 4:**
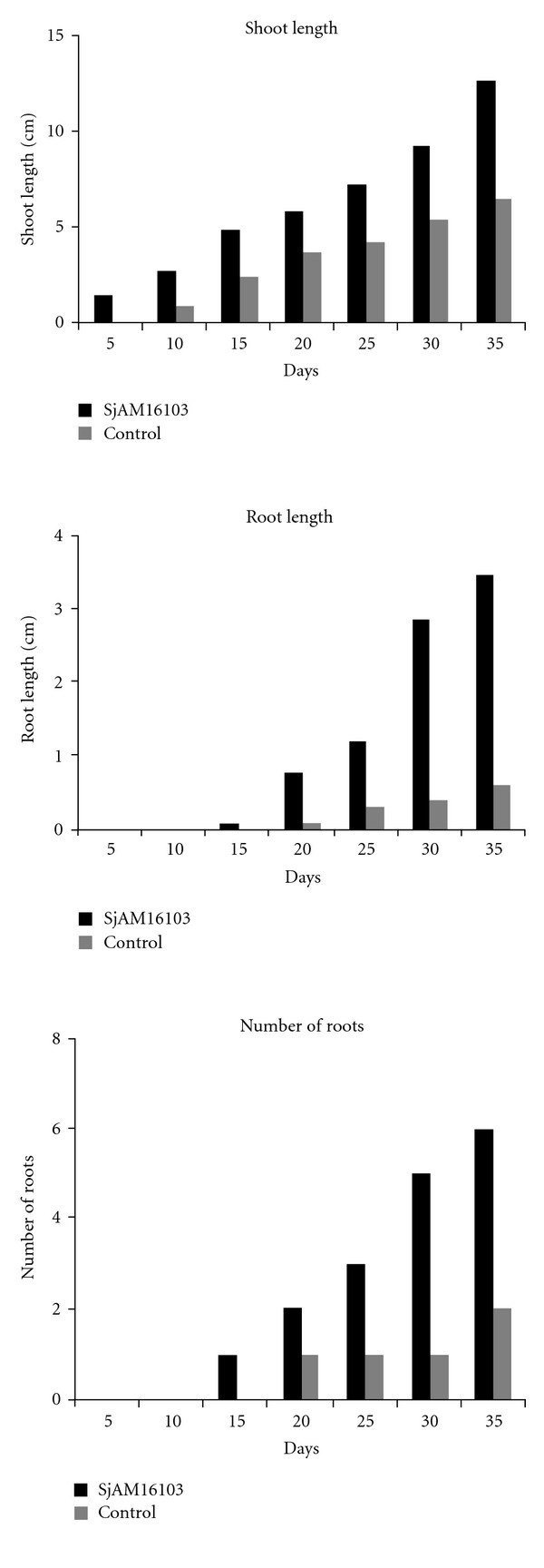
Inoculation of endophytic *S. aquimarina* SjAM16103 in *B*. *monnieri. *

The shoot length and root length of the inoculated explants were increased day by day when compared with control explants. The length of shoot and root of inoculated explants were higher than the control explants 30 days after incubation (Figure 3). Maximum significant (*P* > 0.05) value was observed in the shoot and root length of inoculated explants (Figure 4). Nonsignificant values were observed in control explants. The growth observed in the *S. aquimarina* SjAM16103 inoculated explants was highly significant than the control explants (Table 5). The number of roots counted in the *S. aquimarina* SjAM16103 inoculated explants was highly significant than the control explants (Figure 4).

#### 3.5.2. In *E. triplinerve* Vahl

**Figure 5:**
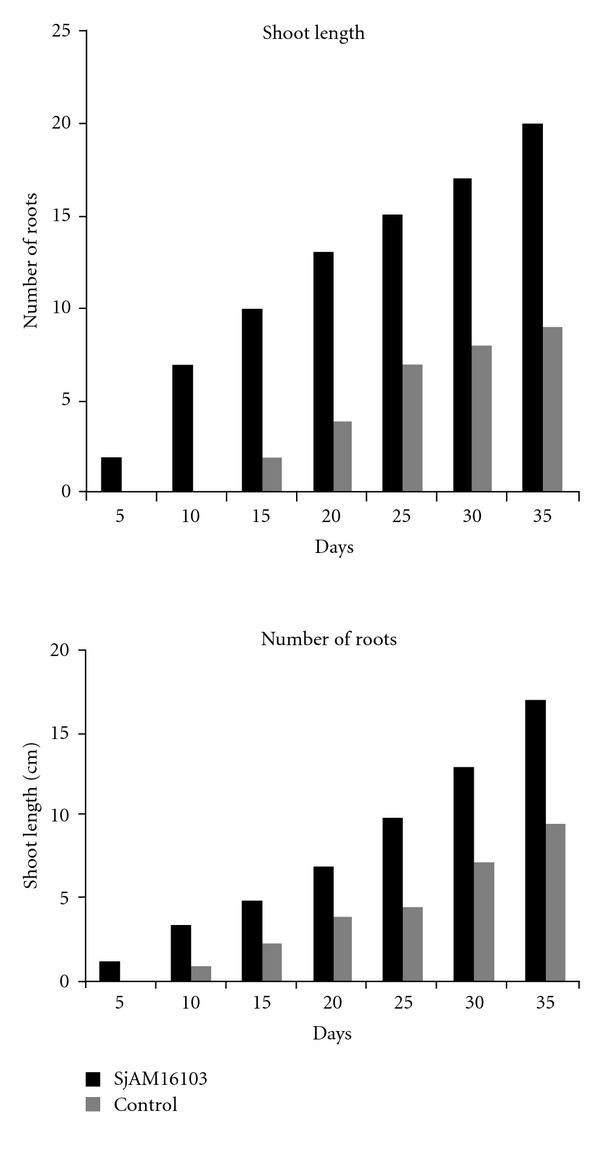
Inoculation of endophytic *S. aquimarina* SjAM16103 in *E*. *triplinerve. *

Growth of shoot in the inoculated explants was increased gradually, and its length was determined 5 days after incubation. The roots were developed after 3 days incubation, and their length was determined 5 days after incubation. The length of shoot and roots of *S. aquimarina* SjAM16103 inoculated explants were higher than the control explants (Figure 5).The shoot and root length of inoculated explants determined were highly significant (*P* > 0.05) than the value of control explants (Table 5). 

**Figure 6:**
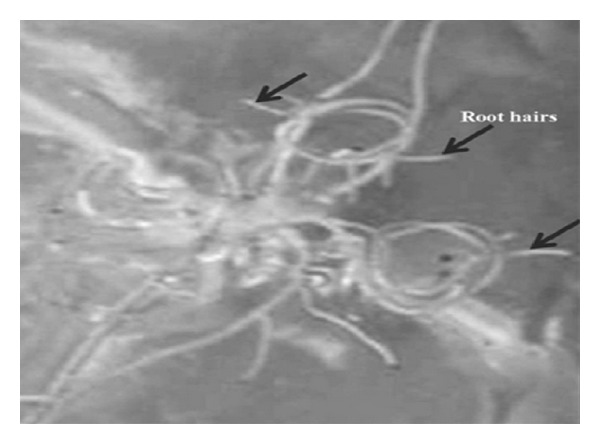
Growth of root hairs in *S. aquimarina* SjAM16103 inoculated explants of *E*. *triplinerve* (*in vitro*).

The numbers of roots were counted with the addition of secondary roots. The numbers of roots of *S. aquimarina* SjAM16103 inoculated explants were highly significant (*P* > 0.05) than the control explants. Root hairs were observed 10 days after incubation in *S. aquimarina* SjAM16103 inoculated explants (Figure 6), whereas root hairs were absent in control explants. This observation provided special concentration for further investigation. 

#### 3.5.3. In *E. agallocha*

**Figure 7:**
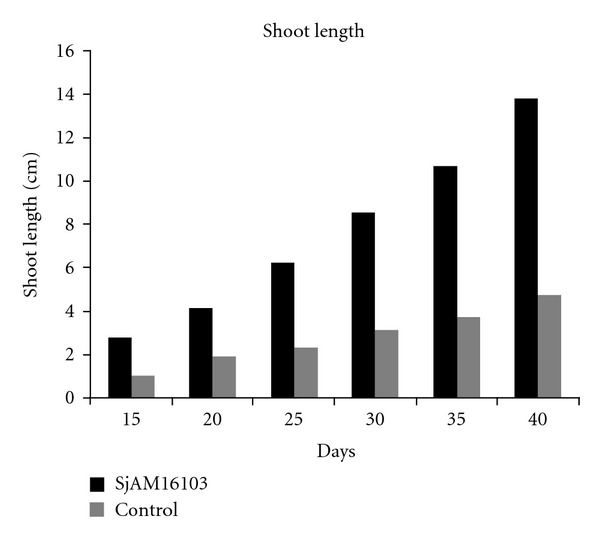
Inoculation of endophytic *S. aquimarina* SjAM16103 in *E*. *agallocha. *

**Figure 8:**
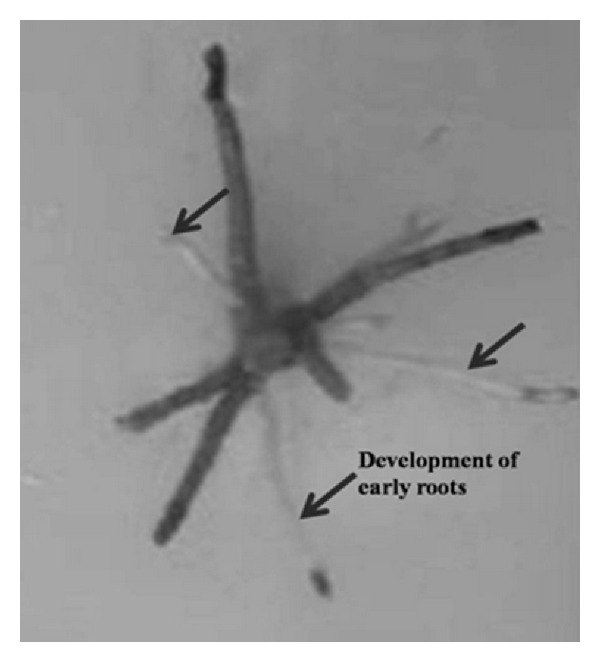
Development of early roots in *S. aquimarina* SjAM16103 inoculated explants of *E*. *agallocha* (*in vitro*).

The shoot length of *S. aquimarina* SjAM16103 inoculated explants, and control explants, were measured 10 days after incubation. Shoot length of inoculated explants was highly significant (*P* > 0.05) than the control explants (Figure 7). Early root development was observed in the *S. aquimarina* SjAM16103 inoculated explants (Figure 8). Roots were developed 15 days after incubation, whereas in control explants roots were developed 30 days after incubation. Root length and number of roots of *S. aquimarina* SjAM16103 inoculated explants were highly significant (*P* > 0.05) than the control explants (Table 5). 

#### 3.5.4. In *A. marina* Vierh

**Figure 9:**
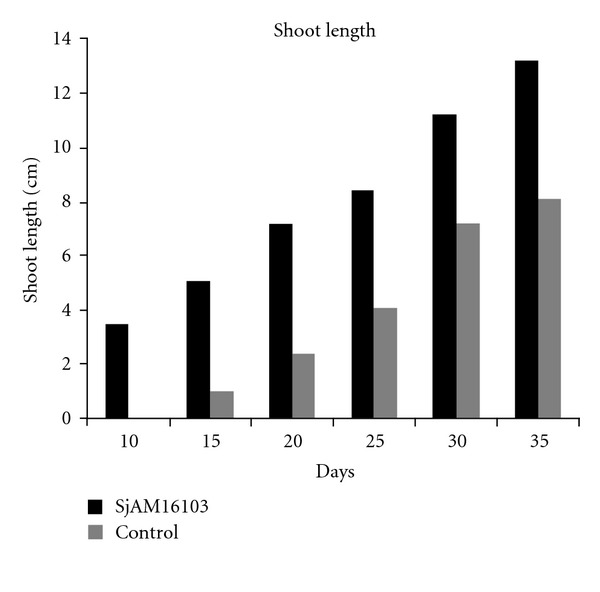
Inoculation of endophytic *S. aquimarina* SjAM16103 in *A*. *marina. *

**Figure 10:**
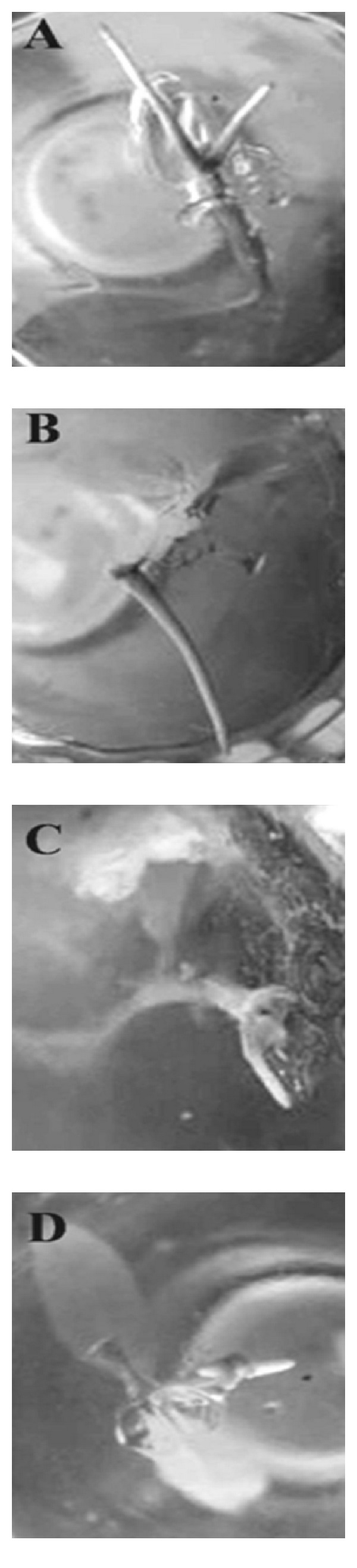
Inoculation of endophytic *S. aquimarina* SjAM16103 in *A*. *marina* (root growth) (*in vitro*). (A and B) Endophytic *S. aquimarina* SjAM16103 inoculated explants; (C and D) control explants.

Shoot growth of *S. aquimarina* SjAM16103 inoculated explants was observed 5 days after incubation and measured 10 days after incubation. Whereas, in control explants, the shoot growth was observed 10 days after incubation and measured 15 days after incubation. Shoot length of *S. aquimarina* SjAM16103 inoculated explants was highly significant (*P* > 0.05) than the control explants (Figure 9). Root growths were observed 20 days after incubation in *S. aquimarina* SjAM16103 inoculated explants (Figure 10). Early leaves were observed in the *S. aquimarina* SjAM16103 inoculated explants. Root length of *S. aquimarina* SjAM16103 inoculated explants was significantly (*P* > 0.05) higher than the root length of control explants (Table 5). 

## 4. Discussion 

Endophytic bacteria are well known in crop plants [9, 32] but largely have not been investigated in mangrove plants. In the present study, thirteen bacterial strains were isolated from the pneumatophores and four strains (SjAM16101, SjAM16102, SjAM16103, and SjAM16104) were selected for further studies based on their morphology and colonization. The strain SjAM16103 was identified as *Sporosarcina aquimarina* using 16s rRNA and was confirmed using biochemical tests and fatty acids profiling. The results obtained in this research are perfectly coincide with reports on endophytes from other hosts in which generally a large number of species can be isolated from a given host, but only very few species are present in a significant number [33]. *S. aquimarina* had been isolated from seawater in Korea [8]. However, this was the first report on *S. aquimarina* isolated from the inner tissue of the plant. 

The strain SjAM16103, *S. aquimarina* could produce siderophore and IAA. Siderophores are iron chelating ligands which can be beneficial to plants by increasing the solubility of ferric iron (Fe III), which otherwise is unavailable for plant nutrition [34]. The production of IAA enhances root growth of the plants by stimulating plant cell elongation or cell division [35]. The colonization of pneumatophores by endophytic *S. aquimarina* SjAM16103 enhances growth of the entire plants. 

The results indicated that reintroduction of naturally occurring endophytic bacteria into tissue culture can lead to improve plant growth and yield. The present study revealed that the endophytic *S. aquimarina* SjAM16103 isolated from the pneumatophores of *A*. *marina* was not host specific. Some endophytic genera, however, exhibit no host specificity and are invariably recovered from plants belonging to different groups and growing in different geographical locations [36, 37].

In the present study, another interesting observation was the growth of root hairs developed in the endophytic *S. aquimarina* SjAM16103 inoculated explants, root hairs could fix atmospheric nitrogen [38]. Endophytic N_2_-fixing bacteria seem to constitute only a small proportion of total endophytic bacteria [39, 40], and increasing N_2_-fixing populations in plants has been considered as a possibility to increase nitrogen fixation. This was the first report on *S. aquimarina* isolated from the living tissue and could produce IAA and fixes atmospheric nitrogen. 

This research has been directed to find endophytic *S. aquimarina* SjAM16103 that could significantly increase the yields in four different plants (*B*. *monnieri*, *E*. *triplinerve*, *E*. *agallocha*, and  *A*. *marina*) after their inoculation. Growth of *S. aquimarina* SjAM16103 inoculated explants of *B*. *monnieri, E*. *triplinerve, E*. *agallocha*, and  *A*. *marina* was highly significant than their control explants. The development of leaves in *S. aquimarina* SjAM16103 inoculated explants was much earlier than control explants. Leaves were developed in *S. aquimarina* SjAM16103 inoculated explants of *A*. *marina* 5 days after incubation, whereas, in control explants, leaves were developed 13 days after incubation. Inoculant seems to be successful in this micropropagated plants, as there were few or no other microorganisms with which to compete. There could be enormous benefits to be gained through the inoculation of microorganisms into soilless mixes in which plants are transplanted at an early stage in their growth. The natural condition of plants seems to be in a close interaction with endophytes. 

Roots were developed earlier in the *S. aquimarina* SjAM16103 inoculated explants than the control explants. *S. aquimarina* SjAM16103 inoculated explants of *E*. *triplinerve* showed the growth of root hairs, whereas this characteristic feature was absent in the control explants of *E*. *triplinerve*. Early roots were observed in the *S. aquimarina* SjAM16103 inoculated explants of *E*. *agallocha*. Endophytic *S. aquimarina* SjAM16103 seems promising to increase crop yields, produced IAA and siderophore, fixed nitrogen, and solubilized phosphate. The distribution and biological activity of this endophytic bacterium deserve to be explored to make full use of their habitation inside the plants. The present study reveals that endophytic *S. aquimarina* SjAM16103 can be used as a biofertilizer, which can subsequently be used by the plant, thereby improving plant growth.

## 5. Conclusion 

In the present study, mangrove was chosen because mangrove ecosystems are known for high productivity. At the same time, pneumatophores were chosen for this research due to its mechanisms (anaerobic respiration), which taken up gases directly from the atmosphere and various other nutrients, like iron, from the inhospitable soil. This report states that endophytic *S. aquimarina* SjAM16103 promotes the plant growth and produces plant growth promoting substances probably by means similar to plant growth-promoting rhizobacteria (PGPR). Plant growth promoting bacteria were environmentally friendly alternative to chemical fertilizers and pesticides, the use of which was regulated and sometimes forbidden, the market for bioinoculants is still expanding. Therefore, a better understanding of endophytic *S. aquimarina* SjAM16103 may help to elucidate its function and potential role more effectively in developing sustainable systems in agricultural field.

## Figures and Tables

**Table 1:** Phenotypic characteristics of *Sporosarcina aquimarina* SjAM16103.

Morphological characteristics	
Shape	Bacilli (with flagellum)
Motility	Motile
Gram's staining	Gram variable
Physiological characteristics	

Temperature	
Min 2–10^°^C	Negative
Max 26–50^°^C	Positive
pH	
4.5	Negative
5.5	Negative
7.0	Positive
9.0	Positive
Salinity	
2%	Positive
3%	Positive
5%	Positive
7%	Positive
9%	Positive

Exoenzyme activities	

Starch hydrolysis	Negative
Gelatin hydrolysis	Positive
Casein hydrolysis	Negative

Endoenzyme activities	

Catalase production	Positive
Urease production	Positive
Oxidase production	Positive
Voges-proskauer	Negative

**Table 2:** Nucleotide sequence of *Sporosarcina aquimarina* SjAM16103.

AAGTGAGGACGCCGCTCTCGCGAAGGTTAAGCTTACTACTTCTTTTGCAACCCACTCCCATGGTGT	
GACGGGCGGTGTGTACAAGGCCCGGGAACGTATTCACCGTGGCATTCTGATCCACGATTACTAGCG	
ATTCCGACTTCATGGAGTCGAGTTGCAGACTCCAATCCGGACTACGACATACTTTATGAGGTCCGC	
TTGCTCTCGCGAGGTCGCTTCTCTTTGTATATGCCATTGTAGCACGTGTGTAGCCCTGGTCGTAAGG	
GCCATGATGACTTGACGTCATCCCCACCTTCCTCCAGTTTATCACTGGCAGTCTCCTTTGAGTTCCC	
GGCCGGACCGCTGGCAACAAAGGATAAGGGTTGCGCTCGTTGCGGGACTTAACCCAACATTTCACA	
ACACGAGCTGACGACAGCCATGCAGCACCTGTCTCACGGTTCCCGAAGGCACTAAGGCATCTCTGC	
CGAATTCCGTGGATGTCAAGACCAGGTAAGGTTCTTCGCGTTGCATCGAATTAAACCACATGCTCC	
ACCGCTTGTGCGGGCCCCCGTCAATTCATTTGAGTTTTAACCTTGCGGCCGTACTCCCCAGGCGGT	
CGACTTAACGCGTTAGCTCCGGAAGCCACGCCTCAAGGGCACAACCTCCAAGTCGACATCGTTTAC	
GGCGTGGACTACCAGGGTATCTAATCCTGTTTGCTCCCCACGCTTTCGCACCTGAGCGTCAGTCTTT	
GTCCAGGGGGCCGCCTTCGCCACCGGTATTCCTCCAGATCTCTACGCATTTCACCGCTACACCTGG	
AATTCTACCCCCCTCTACAAGACTCAAGCCTGCCAGTTTCGAATGCAGTTCCCAGGTTGAGCCC	

**Table 3:** Fatty acid composition of *Sporosarcina aquimarina* SjAM16103.

Saturated fatty acids		Unsaturated fatty acids		Branched fatty acids		Summed feature	
C_10:0_	0.17	C_11:0_ 3OH	0.15	C_13:0_ iso	3.15	1	—
C_12:0_	0.86	C_12:0_ 2OH	0.19	C_13:0_ anteiso	0.14	2	2.87
C_14:0_	2.63	C_12:0_ 3OH	0.41	C_14:0_ iso	—	3	6.40
C_15:0_	—	C_15:0_ 2OH	0.19	C_15:0_ iso	28.39	4	—
C_16:0_	8.99	C_15:0_ 3OH	0.58	C_15:0_ anteiso	0.78	8	9.92
C_17:0_	0.54	C_15:1_ w5	0.19	C_15:0_ iso 3OH	3.00	9	7.25
C_18:0_	0.68	C_15:1_ w6	0.17	C_15:1_ iso F	0.07		
C_19:0_	0.06	C_15:1_ w8	0.08	C_15:1_ anteiso A	—		
		C_16:0_ 3OH	2.15	C_16:0_ iso	1.61		
		C_16:0_ N alcohol	—	C_16:0_ iso 3OH	0.59		
		C_16:1_ w5	0.06	C_16:1_ iso H	—		
		C_16:1_ w7 alcohol	0.25	C_17:0_ iso	0.68		
		C_16:1_ w11	—	C_17:0_ anteiso	0.27		
		C_17:0_ 2OH	0.11	C_17:0_ cyclo	1.42		
		C_17:0_ 3OH	0.13	C_17:0_ iso 3OH	8.96		
		C_17:1_ w6	—	C_17:1_ iso w10	—		
		C_17:1_ w8	0.58	C_17:1_ anteiso w9	—		
		C_18:1_ w9	0.62	C_17:1_ iso w10	—		
		C_20:1_ w7	0.12	C_19:0_ cyclo w8	0.25		
		C_20:1_ w9	—				
Unknown fatty acids		11.543					

**Table 4:** Plant growth-promoting activities of *Sporosarcina aquimarina* SjAM16103.

Growth promoting activities	Result
IAA production	2.37 (*μ*Mol/mL)
P-solubilization	Positive
S-reduction	Negative
N-fixation	Positive
Siderophore production	Positive

IAA: Indole acetic acid production; P: phosphate solubilization; S: sulphur reduction.

**Table 5:** Two-way ANOVA for the differences in shoot length, root length, and number of roots of inoculated and control explants.

Plants	Shoot length	Root length	Number of roots
*B. monnieri* control explants	0.011	−0.034	−0.012
*B. monnieri* inoculated with endophytic bacterium	0.791^∗^	0.556^∗^	0.798^∗^
*E. triplinerve* control explants	0.009	−0.193	−0.174
*E. triplinerve* inoculated with endophytic bacterium	0.907^∗^	0.628^∗^	0.904^∗^
*E. agallocha* control explants	0.010	−0.818	−0.928
*E. agallocha* inoculated with endophytic bacterium	0.936^∗^	0.083^#^	0.258^∗^
*A. marina* control explants	0.003	−0.545	−0.367
*A. marina* inoculated with endophytic bacterium	0.863^∗^	0.074^#^	0.279^∗^

Highly significant (*P* > 0.5) represented by ^∗^ and significant (*P* = 0.5) represented by ^#^.

## Abbreviations

*μ*m: Micrometer
*μ*Mol/mL: Micromoler per milliliter
mol %: Moler percentage
ng: Nanogram
pMol: Pico mole.

## References

[1] Kluyver AJ, van Neil CB (1936). Prospects for a natural classification of bacteria. *Zentbl Bakteriol Parasitenkd Infektkrankh Hyg Abt II*.

[2] Claus D, Fahmy F, Rolf HJ, Tosunoglu N (1983). *Sporosarcina halophila* sp. nov., an obligate, slightly halophilic bacterium from salt marsh soils. *Systematic and Applied Microbiology*.

[3] Claus D, Fahmy F, Sneath PHA, Mair NS, Sharpe ME, Holt J (1986). Genus *Sporosarcina* Kluyver and van Niel (1936) 401AL. *Bergey’s Manual of Systematic Bacteriology*.

[4] Larkin JM, Stokes JL (1967). Taxonomy of psychrophilic strains of *Bacillus*. *Journal of Bacteriology*.

[5] Nakamura LK (1984). *Bacillus psychrophilus* sp. nov., nom. rev. *International Journal of Systematic Bacteriology*.

[6] Miquel P (1889). Étude sur la fermantation ammoniacale et sur les ferments de l’ureé. *Annales de Micrographie*.

[7] Ash C, Farrow JAE, Wallbanks S, Collins MD (1991). Phylogenetic heterogeneity of the genus *Bacillus* revealed by comparative analysis of small-subunit-ribosomal RNA sequences. *Letters in Applied Microbiology*.

[8] Yoon JH, Lee KC, Weiss N, Kho YH, Kang KH, Park YH (2001). *Sporosarcina aquimarina* sp. nov., a bacterium isolated from seawater in Korea, and transfer of *Bacillus globisporus* (larkin and stokes 1967), *Bacillus psychrophilus* (Nakamura 1984) and *Bacillus pasteurii* (Chester 1898) to the genus *Sporosarcina* as *Sporosarcina globispora* comb. nov., *Sporosarcina psychrophila* comb. nov and *Sporosarcina pasteurii* comb. nov., and emended description of the genus. *International Journal of Systematic and Evolutionary Microbiology*.

[9] Hallmann J, Quadt-Hallmann A, Mahaffee WF, Kloepper JW (1997). Bacterial endophytes in agricultural crops. *Canadian Journal of Microbiology*.

[10] Sturz AV, Christie BR, Nowak J (2000). Bacterial endophytes: potential role in developing sustainable systems of crop production. *Critical Reviews in Plant Sciences*.

[11] Boddey RM, Dobereiner J (1988). Nitrogen fixation associated with grasses and cereals: recent results and perspectives for future research. *Plant and Soil*.

[12] Bent E, Tuzun S, Chanway CP, Enebak S (2001). Alterations in plant growth and in root hormone levels of lodgepole pines inoculated with rhizobacteria. *Canadian Journal of Microbiology*.

[13] Reyes I, Bernier L, Antoun H (2002). Rock phosphate solubilization and colonization of maize rhizosphere by wild and genetically modified strains of Penicillium rugulosum. *Microbial Ecology*.

[14] Compant S, Reiter B, Sessitsch A, Nowak J, Clément C, Barka EA (2005). Endophytic colonization of Vitis vinifera L. by plant growth-promoting bacterium Burkholderia sp. strain PsJN. *Applied and Environmental Microbiology*.

[15] Romero D, De Vicente A, Rakotoaly RH (2007). The iturin and fengycin families of lipopeptides are key factors in antagonism of *Bacillus* subtilis toward Podosphaera fusca. *Molecular Plant-Microbe Interactions*.

[16] Nowak J, Pruski K Priming tissue cultured propagules.

[17] Bandaranayake WM (1999). Economic, traditional and medicinal uses of mangroves. *AIMS Report*.

[18] Karalai C, Wiriyachitra P, Opferkuch HJ, Hecker E (1994). Cryptic and free skin irritants of the daphnane and tigliane types in Latex of Excoecaria agallocha. *Planta Medica*.

[19] Ghani A (1998). *Medicinal Plants of Bangladesh: Chemical Constituents and Uses*.

[20] Tripathi YB, Chaurasia S, Tripathi E, Upadhyay A, Dubey GP (1996). Bacopa monniera Linn. as an antioxidant: mechanism of action. *Indian Journal of Experimental Biology*.

[21] Stough C, Lloyd J, Clarke J (2001). The chronic effects of an extract of Bacopa monniera (Brahmi) on cognitive function in healthy human subjects. *Psychopharmacology*.

[22] Gagne S, Richard C, Roussean H, Antoun H (1987). Xylem-residing bacteria in alfalfa roots. *Canadian Journal of Microbiology*.

[23] Gordon RE, Haynes WC, Pang CHN (1973). *The Genus Bacillus. Agriculture Handbook no. 427*.

[24] Sambrook J, Fritsch EF, Maniatis T (1989). *Molecular Cloning, A Laboratory Manual*.

[25] Altschul SF, Madden TL, Schäffer AA (1997). Gapped BLAST and PSI-BLAST: a new generation of protein database search programs. *Nucleic Acids Research*.

[26] Gordon SA, Weber RP (1951). Colorimetric estimation of indoleacetic acid. *Plant Physiology*.

[27] Frankenberger WT, poth M (1988). L-tryptophan transaminase of a bacterium isolated from the rhizosphere of Festuca octoflora (graminae). *Soil Biology and Biochemistry*.

[28] Rao NSS (1999). *Soil Microbiol (Fourth Edition of Soil Microorganisms and Plant Growth)*.

[29] Alexander DB, Zuberer DA (1991). Use of chrome azurol S reagents to evaluate siderophore production by rhizosphere bacteria. *Biology and Fertility of Soils*.

[30] Alderete LM, Mori M, Kato A, Escandón AS (2006). Establishment of an in vitro micropropagation protocol for Mecardonia tenella. *Electronic Journal of Biotechnology*.

[31] Murashige T, Skoog F (1962). A revised medium for rapid growth and bioassays with tobacco tissue cultures. *Plant Physiology*.

[32] Lodewyckx C, Vangronsveld J, Porteous F (2002). Endophytic bacteria and their potential applications. *Critical Reviews in Plant Sciences*.

[33] Petrini O, Sieber TN, Toti L, Viret O (1992). Ecology, metabolite production, and substrate utilization in endophytic fungi. *Natural toxins*.

[34] Renshaw JC, Robson GD, Trinci APJ (2002). Fungal siderophores: structures, functions and applications. *Mycological Research*.

[35] Glick BR, Bashan Y (1997). Genetic manipulation of plant growth-promoting bacteria to enhance biocontrol of phytopathogens. *Biotechnology Advances*.

[36] Sturz AV, Christie BR (1996). Endophytic bacteria of red clover as agents of allelopathic clover- maize syndromes. *Soil Biology and Biochemistry*.

[37] Sturz AV, Christie BR, Matheson BC (1998). Associations of bacterial endophyte populations from red clover and potato crops with potential for beneficial allelopathy. *Canadian Journal of Microbiology*.

[38] Barraquio WL, Revilla L, Ladha JK (1997). Isolation of endophytic diazotrophic bacteria from wetland rice. *Plant and Soil*.

[39] Ladha JK, Barraquio WL, Watanabe I (1983). Isolation and identification of nitrogen fixing *Enterobacter cloacae* and *Klebsiella planticola* associated with rice plants. *Canadian Journal of Microbiology*.

[40] Martínez L, Caballero-Mellado J, Orozco J, Martínez-Romero E (2003). Diazotrophic bacteria associated with banana (Musa spp.). *Plant and Soil*.

